# A Type VI Secretion System Encoding Locus Is Required for *Bordetella bronchiseptica* Immunomodulation and Persistence *In Vivo*

**DOI:** 10.1371/journal.pone.0045892

**Published:** 2012-10-12

**Authors:** Laura S. Weyrich, Olivier Y. Rolin, Sarah J. Muse, Jihye Park, Nicholas Spidale, Mary J. Kennett, Sara E. Hester, Chun Chen, Edward G. Dudley, Eric T. Harvill

**Affiliations:** 1 Department of Veterinary and Biomedical Sciences, The Pennsylvania State University, University Park, Pennsylvania, United States of America; 2 Department of Food Science, The Pennsylvania State University, University Park, Pennsylvania, United States of America; 3 Graduate Program in Biochemistry, Microbiology, and Molecular Biology, The Pennsylvania State University, University Park, Pennsylvania, United States of America; 4 Graduate Program in Immunology and Infectious Disease, The Pennsylvania State University, University Park, Pennsylvania, United States of America; 5 Graduate Program in Bioinformatics, The Pennsylvania State University, University Park, Pennsylvania, United States of America; Institut Pasteur Paris, France

## Abstract

Type VI Secretion Systems (T6SSs) have been identified in numerous Gram-negative pathogens, but the lack of a natural host infection model has limited analysis of T6SS contributions to infection and pathogenesis. Here, we describe disruption of a gene within locus encoding a putative T6SS in *Bordetella bronchiseptica* strain RB50, a respiratory pathogen that circulates in a broad range of mammals, including humans, domestic animals, and mice. The 26 gene locus encoding the *B. bronchiseptica* T6SS contains apparent orthologs to all known core genes and possesses thirteen novel genes. By generating an in frame deletion of *clpV*, which encodes a putative ATPase required for some T6SS-dependent protein secretion, we observe that ClpV contributes to *in vitro* macrophage cytotoxicity while inducing several eukaryotic proteins associated with apoptosis. Additionally, ClpV is required for induction of IL-1β, IL-6, IL-17, and IL-10 production in J774 macrophages infected with RB50. During infections in wild type mice, we determined that ClpV contributes to altered cytokine production, increased pathology, delayed lower respiratory tract clearance, and long term nasal cavity persistence. Together, these results reveal a natural host infection system in which to interrogate T6SS contributions to immunomodulation and pathogenesis.

## Introduction

Highly conserved Type VI Secretion System (T6SS) gene clusters have been recently identified in 92 different strains of bacteria [1]. T6SS loci are disproportionately associated with virulent strains, and multiple virulence-related phenotypes have been attributed to the T6SS in pathogenic bacteria, including mucosal adherence, intracellular growth within macrophages, survival within host cells, and the delivery of bacteriolytic proteins into competitor bacteria [1]–[5]. In *Vibrio cholerae* [6], *Aeromonas hydrophila* [7], and *Legionella pneumophila* [8], T6SS activity enables macrophage cytotoxicity, while T6SSs of *Salmonella typhimurium* and *Yersinia pseudotuberculosis* facilitate HEp-2 cell invasion [9]. Abrogating T6SS functions is associated with reduced virulence *in vivo* of *Aeromonas hydrophila* in a mouse model of septicemia [10], *Pseudomonas aeruginosa* in neutropenic mice [11], *V. cholera* in infant mice and rabbits [12], [13], and *Burkholderia mallei* in hamsters [14]. Strikingly, disruption of the T6SS in Entero-Aggregative *Escherichia coli* (EAEC) does not cause an observable loss of function in a wild type murine infection model [15]. With the exception of *A. hydrophila*, *Salmonella enterica,* and *Francisella tularensis*, many T6SS-associated *in vitro* phenotypes were not observed in adult, wild type mice [7], [16], [17]. Despite evidence that the T6SS enables virulence in multiple species, many of the discrete, *in vivo* interactions between the T6SS and host immunity have not yet been determined.

This study examines the T6SS in the common respiratory pathogen, *Bordetella bronchiseptica.* This Gram-negative bacterium infects a wide range of mammals, including humans, and causes disease severities ranging from asymptomatic carriage to fatal pneumonia. *B. bronchiseptica* commonly causes kennel cough in domesticated animals, snuffles in rabbits, and atrophic rhinitis in swine and is considered the evolutionary progenitor-like strain of *B. pertussis* and *B. parapertussis*, causative agents of whooping cough in humans [18]. *B. bronchiseptica* also efficiently infects and causes disease in laboratory animals, such as mice, rats, and rabbits, providing a natural host infection model that has been used to reveal important interactions between bacterial virulence factors and the host immune system *in vivo* [19], [20].

A considerable number of specific bordetellae virulence determinants, such as autotransporters, adhesins, and toxins, require secretion through various machineries, such as the Type I, Type II, Type III, Type IV, and Type V secretion systems (TnSS) [18], [21]. These secretion systems export factors that enable host epithelium adherence [22], disable the mucociliary escalator [23], manipulate signaling pathways in antigen presenting cells [24], [25], and block neutrophil chemokine receptors [26]. *Bordetella* virulence factors, such as adenylate cyclase toxin (ACT), pertussis toxin (PTX), fimbria, *Bordetella* resistance to killing protein (BrkA), filamentous hemagglutinin (FHA), pertactin (PRN), and tracheal colonization factor (TCF), have all been shown to require secretion systems for export [21], [27]–[29]. Even when many secreted factors are unknown, abrogating secretion by these systems can result in observable effects [24], [30], [31]. For example, increased expression of the *B. bronchiseptica* T3SS locus correlated with hypervirulence *in vivo*, and before a specific secreted effector was identified, disruption of the T3SS was associated with decreased *in vitro* cytotoxicity and *in vivo* pathology [32]–[35]. Although a locus homologous to known T6SSs was not identified in *B. pertussis*, a putative T6SS locus was identified in *B. bronchiseptica* and *B. parapertussis* genomes, and its secreted effectors, function, and contributions to *Bordetella* pathogenesis have not yet been characterized [21], [36].

To examine the role of the T6SS in *Bordetella* pathogenesis, we analyzed the 26 gene locus in *B. bronchiseptica* strain RB50, a strain which has been extensively characterized in various animal models. An in-frame deletion of the gene encoding a putative T6SS ATPase, *clpV*, altered interactions with macrophages *in vitro*, affecting the secretion of IL-1β, IL-6, IL-10 and IL-17. The RB50Δ*clpV* strain was also defective in cytotoxicity toward macrophages i*n vitro*, a phenotype previously associated with both Adenylate Cyclase Toxin (ACT) and a Type Three Secretion System (T3SS). Furthermore, mutation of *hcp*, a structural component of other T6SSs, and a *clpV* mutation in another hypervirulent *B. bronchiseptica* lineage also resulted in a loss of cytotoxicity. During infection in wild type mice, *clpV* was required to induce significant pathology in the lungs. RB50Δ*clpV* was also rapidly cleared from the lower respiratory tract and deficient in nasal cavity persistence. Together, these data indicate that the T6SS plays an essential role in *B. bronchiseptica* pathogenesis and reveal interactions through which the T6SS mediates virulence *in vivo*.

## Materials and Methods

### Ethics Statement

This study was carried out in strict accordance with the recommendations in the Guide for the Care and Use of Laboratory Animals of the National Institutes of Health. The protocol was approved by the Institutional Animal Care and Use Committee at The Pennsylvania State University at University Park, PA (#31297 Bordetella-host Interaction). All animals were anesthetized using isoflourane or euthanized using carbon dioxide inhalation to minimize animal suffering.

### Comparative protein sequence analysis

Based on Boyer *et al.* analysis, there are 35 genes (BB0787–BB0821) in the *B. bronchiseptica* T6SS locus [1]. However, six genes (BB0787–BB0792) upstream of BB0793 were annotated as possible T2SS locus in RB50, and there are only three predicted operons (BB0793–BB0810, BB0811–BB0812, and BB0813–BB0818) within this locus based on OperonDB (http://operondb.cbcb.umd.edu/cgi-bin/operondb/pairs.cgi?genome_id=120). Thus, we have defined the T6SS locus with 26 genes (BB0793–BB0818). The DNA and protein sequences corresponding to all the genes present in T6SS locus of *B. bronchiseptica* strain RB50 were obtained online (http://www.ncbi.nlm.nih.gov); the orthologous genes in *P. aeruginosa, S. enterica,* and *V. cholerae* were located via KEGG ortholog database (http://www.genome.jp/kegg/genes.html). The amino acid sequence similarity was determined by comparing RB50 genes to orthologous genes in *P. aeruginosa, S. enterica,* and *V. cholerae* using the online NCBI protein BLAST search (http://www.ncbi.nlm.nih.gov/BLAST).

### Bacterial strains and growth

*B. bronchiseptica* strain RB50 and strain 1289 have been described elsewhere [35], [37]. Bacteria were maintained on Bordet-Gengou agar (Difco) supplemented with 10% sheep blood (Hema Resources) with 20 µg/ml streptomycin (Sigma). Bacteria were grown in liquid culture to mid-log phase while shaking in Stainer-Scholte (SS) broth [38] overnight at 37°C.

### Construction of RB50Δ*clpV* and 1289Δ*clpV* strains

The RB50Δ*clpV* strain was constructed using an allelic exchange strategy as previously described [35]. The first three codons of *clpV* (BB0810) and the 630 base pairs (bp) upstream were amplified via PCR using primers flanked with EcoRI on the 5′ end and HindIII on the 3′ end (Table S1, 5′F and 5′R). The last eight codons of *clpV* and the 432 bp downstream were amplified via PCR using primers flanked with HindIII on the 5′ end and EcoRI on the 3′ end (Table S1, 3′F and 3′R). These fragments were PCR purified (Qiagen, Valencia, CA), BamHI digested (New England Biolabs), gel purified (Qiagen, Valencia, CA), and ligated overnight at 4°C (New England Biolabs), and amplified with the 5′ F and 3′ R primers as described above. The 1,280 bp knock-out construct was then ligated into the TOPO-TA vector, transformed into Mach1 DH5α cells (Invitrogen), and verified by sequencing. The 1280-bp construct was digested from TOPO-TA, gel purified, and ligated overnight into the EcoRI-digested pSS4245, a *Bordetella* allelic exchange vector (courtesy of S. Stibitz). Triparental mating with DH5α harboring pSS4545 Δ*clpV*, DH5α containing pSS1827, and *B. bronchiseptica* strain grown under Bvg^−^ conditions by growth on BG plus 50 mM MgSO_4_ was done for 4 hrs on a BG-10 mM MgCl_2_-50 mM MgSO_4_ plate at 37°C. Then, *B. bronchiseptica* containing pSS4245 Δ*clpV* was positively selected by growth on BG-streptomycin-kanamycin-50 mM MgSO_4_ plates and incubated for 2 days at 37°C; this step was repeated to ensure purity. The resulting colonies were streaked onto BG plates and incubated for 2 days at 37°C, which resulted in colonies lacking pSS4245 and containing either the wild-type or knockout gene. Colonies were then screened for the presence of either the wild-type or knockout gene by using screening primers (Table S1) which detected either the wild-type *clpV* (2,003 bp) or the Δ*clpV* deletion (1,280 bp) with PCR. The absence of pSS4245 was confirmed by growth on BG-streptomycin plates and lack of growth on BG-kanamycin plates.

### qRT-PCR

Quantitative reverse transcription PCR (qRT-PCR) was preformed as previously described [35], [39], [40]. Briefly, bacteria at an OD_600_ 0.2 were subcultured into four independent five mL cultures until OD_600_ reached 0.8; 10^8^ cells were immediately pelleted by centrifugation at 4°C 8,000 RPM for five minutes. Total RNA was extracted with Trizol (Invitrogen), treated with RNase-free DNase I (Invitrogen), and purified using RNeasy columns (Qiagen) according to the instructions of the manufacturer. One microgram of RNA from each biological replicate was reverse transcribed using 300 ng of random oligonucleotide hexamers and SuperScript III RTase (Invitrogen). The resulting cDNA was then diluted 1∶100, and 1 µL aliquots were used for qRT-PCR. 300 nM of primers (Table S1B) designed using IDT DNA software (http://www.idtdna.com) were used in conjunction with 2× SYBR green PCR master mix (Applied Biosystems). Control samples of reaction mixtures excluding reverse transcriptase were included to confirm the absence of DNA contamination; amplification of the 16S RNA amplicon was used as an internal qRT-PCR control. Dissociation curve analysis was preformed to confirm sample homogeneity. Threshold fluorescence was established within the geometric phase of exponential amplification, and the cycle threshold (*C_T_*) was determined for each sample. The *C_T_* from each replicate was averaged, and the 16S RNA amplicon was used as internal control for data normalization. The change in transcript level was determined using the relative quantitative *C_T_* method (ΔΔ*C_T_*) [41]. All primers used in qRT-PCR analysis can be found in Table S1B.

### Cytotoxicity assay

Cytotoxicity assays were preformed as previously described [34], [35], [42]. Briefly, murine macrophage J774A.1 cells (ATCC) were cultured in Dulbecco's modified Eagle's medium (DMEM, Difco) supplemented with 10% fetal bovine serum, 1% penicillin-streptomycin, 1% nonessential amino acids, and 1% sodium pyruvate. The cells were grown to 85% confluency in 5% CO_2_ in 96-well plates (Greiner Bio-One) at 37°C. DMEM was then replaced with RPMI medium lacking phenol red with 5% fetal bovine serum, 1% L-glutamine, 1% nonessential amino acids, and 1% sodium pyruvate at least one hour prior. Bacteria diluted in RPMI at multiplicities of infection (MOI) of 0.1, 1, and 10 were centrifuged onto the macrophages at 300×g for 5 minutes and incubated in 5% CO_2_ at 37°C for 2, 4, and 6 hours. The cell culture supernatants were collected, and lactate dehydrogenase (LDH) release, a measure of cytotoxicity, was analyzed using a Cytotox96 kit (Promega) according to the instructions of the manufacturer.

### Protein Extraction and 2D Gel Electrophoresis

RAW 264.7 cells obtained from ATCC were grown in DMEM supplemented with 10% FBS in a 5% CO_2_ incubator at 37°C. The cells were grown in a monolayer in 6 well cell culture plates (70% confluency), and serum free media was applied 3 hours before the beginning of the assay. Six wells of monocyte cells were treated with media alone, *B. bronchiseptica* strain RB50, or RB50Δ*clpV* at an MOI of 10, centrifuged at 250×g for 5 minutes, and incubated at 37°C and 5% CO_2_ for 2 hours. Cellular protein was extracted using previously established methods [43]. Briefly, the supernatant was removed from each well, and the cultured cells were washed twice in phosphate-buffered saline (PBS). The cells were then harvested by scraping into 3 mL cold buffer containing 50 mM Tris pH 8.6, 10 mM EDTA, 65 mM DTT, protease-inhibitor cocktail (Pierce), 2000 U/mL DNase I (Ambion) and 2.5 mg/mL RNase A (Qiagen), and the cellular suspensions were pooled. The cells were lysed using a homogenizer at 4°C and centrifuged at 1000×g to remove membranes. The protein concentration was determined using the Pierce 660nm assay (Thermo Scientific), as per manufacturer's instructions. Two-dimensional (2D) electrophoresis was performed using the Ready-Prep 2D Starter Kit (Bio-Rad) using IPG strips with pH range 3–10 (Bio-Rad) for the first dimension and Criterion 12.5% Tris HCL precast gels (Bio-Rad). 500 µg of protein were loaded for each sample, and the gels were stained with Gelcode Blue reagent (Pierce). The gels were analyzed using PDQuest software (Biorad). Protein spots were excised and trypsin digested for analysis using nano-LC MS/MS (Waters QTOF Premier). The proteins were identified using MASCOT software (Matrix).

### Intracellular staining

Intracellular staining of J774 murine macrophages was performed as previously described [44]. Briefly, cells grown on coverslips were washing three times with PBS and fixed in 4% paraformaldehyde in phosphate-buffered saline buffer (PBS) (Omnipur) for ten minutes. Cells were then against washed three times with PBS and blocked with 3% bovine serum albumin in PBS for 30 minutes. The primary antibody, Annexin V-FITC (BD Pharmingen), was diluted in 3% BSA and PBS and incubated with the cells for 1 hour at room temperature. After three washes in PBS, the cells were stained with DAPI/PBS for 10 minutes at room temperature. Cells were then mounted onto glass slides in Vectashield (Vector Laboratories, Inc., Burlingame, CA) and examined using a materials microscope (Olympus BX61) at the Cytometry Core Facility at University Park, PA. All imaged were saved as TIFF files and processed in Microsoft Powerpoint.

### Cytokine detection

Cytokine analysis was preformed as previously described [45], [46]. Briefly, cell culture supernatants were collected from J774 macrophages that were stimulated with RB50 or RB50Δ*clpV* at an MOI of 0.1 for 2, 4, 6, and 24 hours or murine lung homogenates that were used for bacterial quantification and frozen at −80°C until assayed were collected. Interleukin-1β (IL-1β), interleukin-6 (IL-6), interleukin-10 (IL-10), interleukin-17 (IL-17), Interferon-γ (IFN-γ), and tumor necrosis factor α (TNFα) concentrations were determined via ELISA in accordance with the supplier's protocols (R&D Systems).

### Animal experiments

Wild type C57BL/6 mice were obtained from Jackson Laboratories, Bar Harbor, ME. Mice were bred and maintained at a specific pathogen-free facility at The Pennsylvania State University, University Park, PA, and all experiments were carried out in accordance with all institutional guidelines. All animal experiments were done as previously described [20], [35], [47]. Briefly, the number of bacterial colony forming units in liquid cultures was calculated based on the optical density measured by absorbance of light at 600 nm. Bacteria were then diluted to 10^7^ CFU/ml in sterile PBS. Inocula were confirmed by plating dilutions on BG agar and counting the resulting colonies after two days of growth at 37°C. For inoculation, mice were sedated with 5% isoflurane (IsoFlo, Abbott Laboratories) in oxygen and inoculated by gently pipetteing 50 µl PBS containing the indicated CFU of bacteria onto the external nares. For quantification of bacterial numbers, mice were euthanized with CO_2_ inhalation and the indicated organs were excised. Tissues were homogenized in PBS, serially diluted and plated onto BG agar plates with 20 µg/mL streptomycin, and colonies were counted after 2 days of growth at 37°C. Survival curves were generated as previously described [35]. Mice were observed over a 28 day period; any mouse exhibiting lethal bordetellosis, indicated by ruffled fur, labored breathing, and diminished responsiveness, was euthanized immediately to prevent unnecessary suffering [20], [48].

### Lung pathology

Three days following inoculation with either RB50 or RB50Δ*clpV,* the mice were euthanized and the trachea and lungs were inflated with 1.5 ml of 10% formalin in PBS. The tissues were processed and stained with hematoxylin and eosin (H&E) at the Animal Diagnostic Laboratory at The Pennsylvania State University, in University Park, PA. Sections were analyzed and scored on a qualitative scale as previously described [48]. An assessment of microscopic lesions was made by a by one of the authors (M. J. Kennett) experienced in rodent pathology and blinded to experimental treatment. Descriptive evaluations of the lesions were recorded, and lung lesions were graded by using a scale of 0 to 5. Sections with no lesions and no inflammation were given a score of 0, a score of 1 indicated slight inflammation with few or scattered lesions and fewer than 10% of lung fields affected, a score of 2 indicated mild lesions with 10 to 20% of lung fields affected, a score of 3 indicated moderate lesions with 20 to 30% of the lung fields affected, and those given a score of 4 were characterized by extensive lesions, marked inflammation, and 31 to 50% of the lung was affected. A score of 5 indicated there were extensive lesions with >50% of the lung fields affected.

## Results

### T6SS locus in *Bordetella bronchiseptica* strain RB50

**Figure 1:**
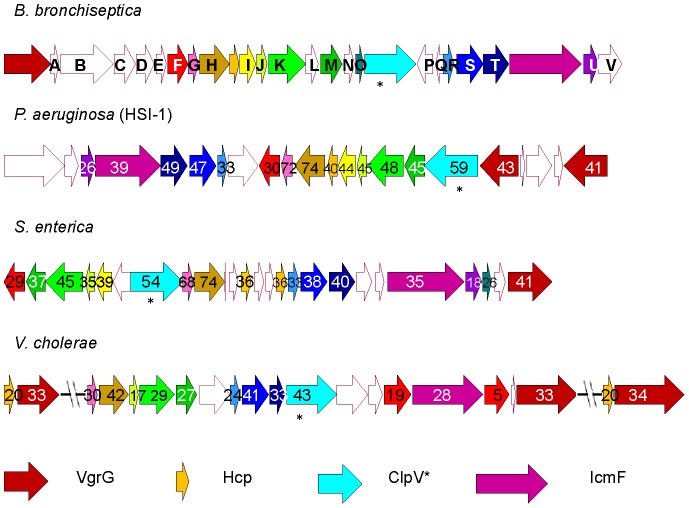
A genetic comparison of *B. bronchiseptica* T6SS locus to known T6SS loci. The T6SS locus from *B. bronchiseptica* is compared to loci in *P. aeruginosa, S. enterica,* and *V. cholerae*. Homologous genes are indicated with the same color, while genes with no homologues are indicated with white color. The numbers in the arrows indicate the percentages of amino acid sequence similarity compared to *B. bronchiseptica.* The length of arrows is relative to the length of the gene. * Indicates the gene targeted for deletion in *B. bronchiseptica* and its homologues.

In 2008, Bingle *et al.* used comparative sequence analysis to first identify the T6SS associated genes present in the published genome of *B. bronchiseptica* [36]. Four of these proteins in *B. bronchiseptica* shared homology to proteins that have been dubbed the ‘core components’ of the T6SS machinery: ClpV, IcmF, Hcp, and VgrG [1], [36]. In this study, we characterize a contiguous 26 gene locus in *B. bronchiseptica* strain RB50 predicted to encode proteins sharing high amino acid sequence similarity with highly conserved T6SS proteins found in *V. cholerae, S. enterica* and *P. aeruginosa* (Figure 1). We have retained the names of the T6SS ‘core component’ genes, while naming the unique T6SS genes of *Bordetella tssA-V*, representing “type six secretion” and maintaining nomenclature put forth by Shalom, *et al* (Figure 1) [49]. Amino acid sequence motif analysis of the predicted protein ClpV, a putative AAA^+^ ATPase, revealed two ATP binding sites with Walker A and B motifs [50] (J. Park and E.T. Harvill, unpublished data), suggesting that it may enable effector molecule binding or provide energy for protein translocation, as observed in *V. cholerae, P. aeruginosa*, *E. coli,* and other bacteria [36], [51]. Recently, Basler *et al.* indicated that *clpV* was not essential for all of the T6SS-dependent bacteriocidal activities in *V. cholerae* and may be required for the retraction of the contractile sheath-like structure of the T6SS [52], [53], indicating that ClpV may contribute to cellular activities in addition to its predicted ATPase functions. The predicted IcmF protein identified in bordetellae is similar to the IcmF- and IcmH-like proteins found in the T4SS. Yeast two hybrid assays in *Edwardsiella tarda* suggest that IcmF- and IcmH-like proteins may form a transport apparatus and act synergistically in translocating substrates [54]. A gene sharing 20% amino acid sequence identity to the Hcp encoding gene in *V. cholerae* and 40% identity with that of *P. aeruginosa* was identified in *B. bronchiseptica*. Hcp may function as an effector and/or assemble into a hexameric ring structure that forms a channel or pilus for conduction of other effectors through the cell membrane, which has been shown to be essential for T6SS mediated virulence in *Vibrio* and *Pseudomonas* [55]. A homolog of *vgrG*, currently annotated as *vrgS*, was identified in *B. bronchiseptica*, possessing 34% and 41% sequence identity with *V. cholerae* and *P. aeruginosa vgrG* genes, respectively, and contains the GP5 region predicted to form the base of the needle apparatus. VgrG of *V. cholerae* contains regions of homology to the actin cross-linking domain of an RtxA toxin [56], and more recently has been shown to share homology with the gp27 of T4 bacteriophage, which forms part of a tail spike apparatus for membrane penetration [57], [58]. Both Hcp and VgrG are found in the secretomes of most bacteria possessing a functional T6SS, even though they lack an export signal peptide [4], [6], [36]. These same four ‘core’ components of the T6SS machinery were also identified in *B. parapertussis* strain 12822; however, further DNA sequence analysis of this locus identified frameshift mutations in upstream genes and a pseudogene that replaced *vgrG* (J. Park and E.T. Harvill, unpublished data), suggesting it may be either defective or functionally different from the locus found in *B. bronchiseptica*. No T6SS genes were found in the sequenced genome of *B. pertussis* strain Tohoma I, indicating that this locus may have been lost through genome degradation in the course of *B. pertussis* evolution [59].

### Deletion of *clpV* from *B. bronchiseptica* strain RB50

**Figure 2:**
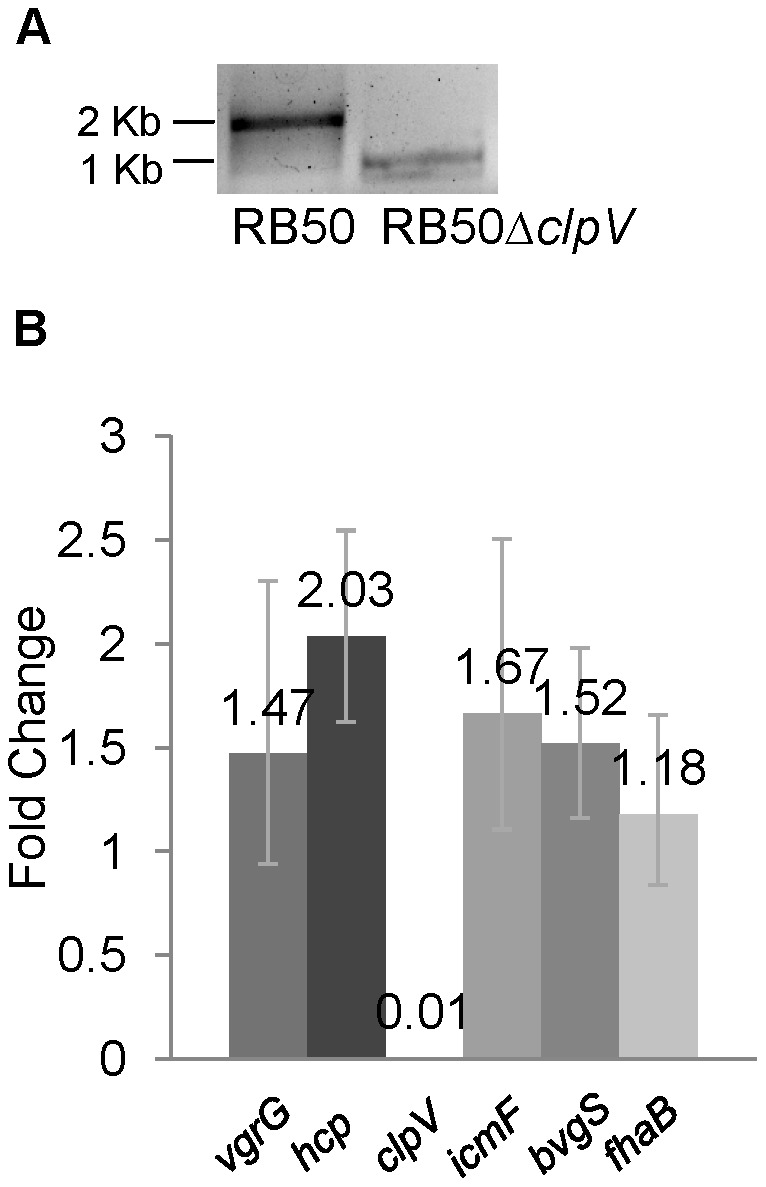
Confirmation of RB50Δ*clpV* construction by PCR and RT-PCR analysis. A. PCR analysis of *clpV* in RB50 (left) and RB50Δ*clpV* (right). Size markers are designated on the left. B. RT-PCR analysis of relative expression of *vgrG, hcp, clpV, icmF, bvgS,* and *fhaB* in RB50Δ*clpV* relative to RB50 expressed as mean ± standard deviation. Each gene was normalized to the expression of 16S RNA.

ClpV has been shown to be required for translocation of T6SS effector proteins essential for virulence in *V. cholerae, P. aeruginosa*, and *E. coli* [3], [4], [60]. To investigate the role of T6SS in *B. bronchiseptica* pathogenesis, we constructed an in-frame deletion of *clpV* (BB0810) in the genome of *B. bronchiseptica* strain RB50 (RB50Δ*clpV*) by utilizing the bordetellae allelic exchange vector pSS4245 [35]. The wild type gene produced a 2,003 bp PCR product, whereas the *clpV* mutant region resulted in a product of 1,280 bp, as expected (Figure 2A). Following deletion of *clpV*, expression of the four core T6SS proteins, *icmF*, *hcp*, *vgrG*, and *clpV* in RB50Δ*clpV* was compared with expression in the parental strain by qRT-PCR. Expression of *icmF*, *hcp*, and *vgrG* in the mutant strain remained comparable to that of RB50 while *clpV* expression was reduced to background levels, suggesting that this gene has been effectively disrupted without altering the expression of neighboring genes (Figure 2B). The growth rate of RB50Δ*clpV* in SS broth at 37°C was not different from that of parental strain RB50 (data not shown), further suggesting that *clpV* was successfully disrupted without causing additional defects.

### T6SS contributes to cytotoxicity of murine macrophages

**Figure 3:**
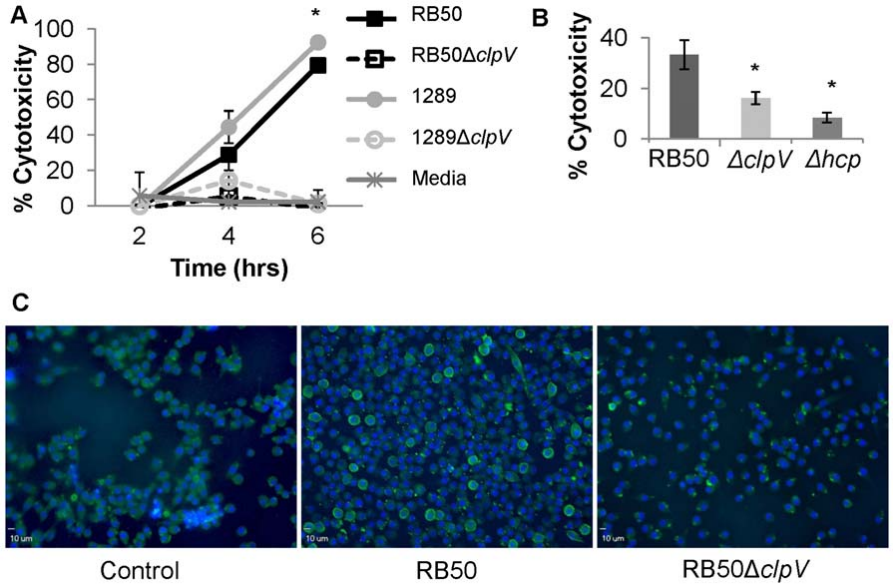
T6SS mediates cytotoxicity in murine macrophages. A–B. LDH release assay monitoring cytotoxicity of J774 murine macrophages at an MOI of 1 for 2, 4, and 6 hour incubations with RB50, RB50Δ*clpV*, 1289, or 1289Δ*clpV* (A), and the same assay conducted independently to compare RB50, RB50Δ*clpV*, and RB50Δ*hcp* induced cytotoxicity after a 4 hour incubation (B). C. J774 macrophages stained with Annexin V (green) and DAPI (blue) after incubation with RB50, RB50Δ*clpV*, or media alone for three hours. * denotes p value<0.05.

In other bacterial systems, the T6SS has been found to mediate interactions between bacteria and phagocytic cells, including protection against amoeba predation, enhanced intracellular survival within macrophages, and the ability to directly kill macrophages *in vitro* [4], [6], [7], [16]. Additionally, because *B. bronchiseptica* is known to be highly cytotoxic to cultured macrophages, we hypothesized that the T6SS may play a role in cytotoxicity. Using a lactate dehydrogenase (LDH) release assay, we measured the cytotoxic effects of *B. bronchiseptica* on murine macrophages. When macrophages were infected with either RB50 at a MOI of 1, we observed increasing levels of cytotoxicity over the first 6 hours of incubation (Figure 3A). By six hours, 71% of macrophages were lysed by RB50, as previously described [35], [61], [62]. Minimal cytotoxicity was observed in macrophages exposed to RB50Δ*clpV* at any point throughout the 6 hour incubation (Figure 3A). Because complementation by plasmid expression of *clpV* was unsuccessful after multiple attempts, *clpV* was deleted from RB50 three times, independently, with identical effects on cytotoxicity (data not shown). Further, we deleted *clpV* from another wild-type *B. bronchiseptica* isolate, strain 1289, known to exhibit hypervirulence and increased cytotoxicity associated with T3SS overexpression. As expected, wild type 1289 induced 92% cytotoxicity after six hours incubation, more than the 71% induced by RB50 (Figure 3A). Similar to RB50Δ*clpV*, the *clpV* mutant of 1289 induced minimal cytotoxicity at all time points, suggesting that ClpV is required for macrophage killing in multiple *B. bronchiseptica* strains. A mutant lacking the gene encoding Hcp, which encodes a putative T6SS structural component, was additionally constructed in our laboratory (S. J. Muse and E. T. Harvill, unpublished data) and also failed to induce cytotoxicity (Figure 3B), further supporting a role for the *B. bronchiseptica* T6SS in macrophage killing.

To examine the ClpV-mediated mechanism of LDH release, J774 cells were stained for the presence of Annexin V after being incubated with either strain RB50 or RB50Δ*clpV* at an MOI of 100 for 2 hours. Significantly more Annexin V positive macrophages were observed when cells were incubated with RB50 compared to RB50Δ*clpV* (Figure 3C). Additionally, RB50Δ*clpV* stimulated small Annexin V puncta near the membrane without stimulating full Annexin V membrane staining. Together, these results suggest that the T6SS contributes to the apoptotic death of macrophages *in vitro*.

### Macrophage proteome changes in response to T6SS

**Figure 4:**
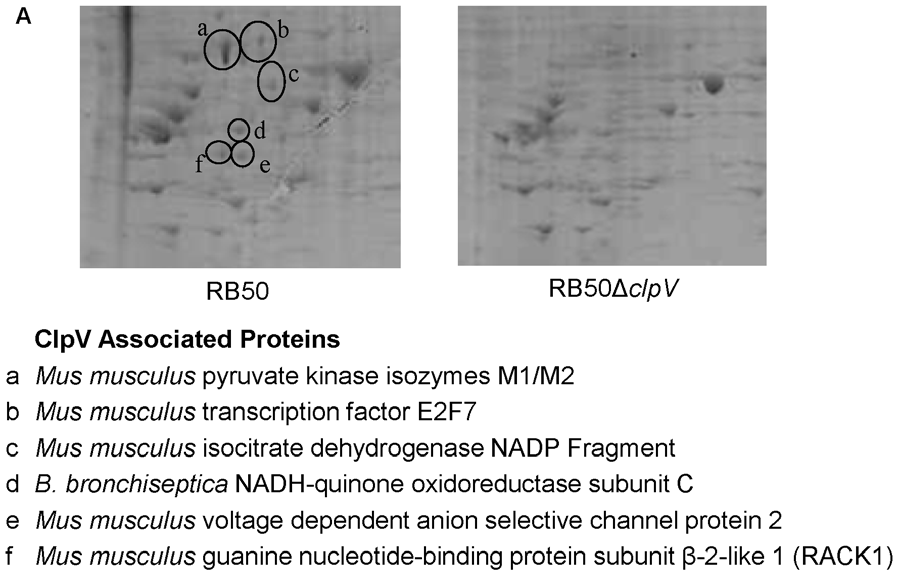
T6SS induces changes in the macrophage proteome during *in vitro* infection. A. Two dimensional gel electrophoresis was performed on whole cell extract from macrophages infected with either RB50 or RB50Δ*clpV* at an MOI of 10 for 2 hours. A section of the 2D gel image is shown, and the entire 2D gel image can be seen in Figure S1. The six most prominent proteins, which were also selected for identification by mass spectrometry, are circled in black and labeled (a–e). Top hits from MASCOT correlating to each identified protein (a–e) are listed below the images.

To investigate which cell signaling pathways are involved in T6SS-mediated macrophage cell death, we analyzed the proteome of murine macrophages exposed to either RB50 or RB50Δ*clpV*. Murine J774 macrophages were stimulated with either RB50 or RB50Δ*clpV* at an MOI of 10 for 2 hours, lysed, and analyzed by two dimensional (2D) gel electrophoresis. This approach enables identification of bacterial proteins secreted into eukaryotic cells in a contact dependent manner [4]. This approach also enables detection of proteins that are differentially produced by macrophages in response to a functional T6SS. A total of 431 different proteins were visualized by 2D gel electrophoresis, and 283 proteins differed between RB50 or RB50Δ*clpV* infected macrophages (Figure S1 and S2). The six most prominent proteins that were only observed in RB50 infected cells (proteins a–e) were identified via mass spectrometry as an initial analysis to investigate how murine macrophages are affected by the *B. bronchiseptica* T6SS (Figure 4A). Murine macrophage proteins identified included pyruvate kinase isoxyme M1 M2, transcription factor E2F7, isocitrate dehydrogenase NADP Fragment, voltage dependent anion selective channel protein 2, and guanine nucleotide binding protein subunit beta 2 like 1. NADH-quinone oxidoreductase subunit C was the only bacterial protein identified. Together, this data suggests that deletion of *clpV* changes the macrophage cellular response produced during infection *in vitro*.

### T6SS stimulates IL-1β and IL-6 production *in vivo*

**Figure 5:**
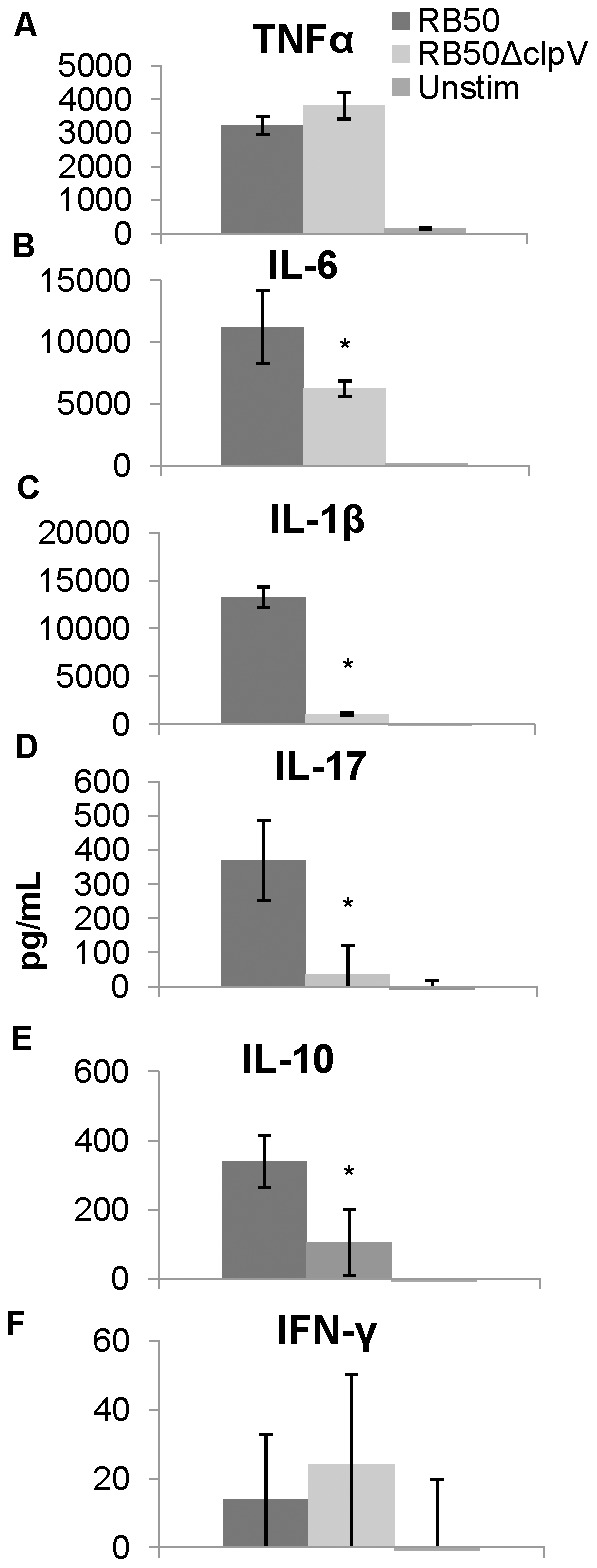
ClpV contributes to cytokine production *in vitro*. A–C. Supernatants from infected murine macrophages were recovered after 24 hours at an MOI of 0.1 of RB50 (dark bars) or RB50Δ*clpV* (light bars) and assayed for TNFα (A), IL-6 (B), IL-1β (C), IL-17 (D), IL-10 (E) and IFN-γ (F). * denotes p value<0.05.

To assess the effects of *clpV* deletion on macrophage cytokine production, cultured macrophages were exposed to either RB50 or RB50*ΔclpV* at an MOI of 0.1 for 24 hours, and IL-1β, IL-6, IL-10, IL-17, IFN-γ, and TNFα were analyzed because these cytokines are known to contribute to host immunity or pathogenesis [45], [48]. No changes were observed in TNFα production at 2 hours post inoculation (data not shown). By 24 hours of stimulation with either wild type or the mutant bacteria, similar amounts of TNFα were produced (Figure 5A). In contrast, macrophages exposed to RB50 produced more IL-6 and IL-1β than those exposed to RB50Δ*clpV* (Figure 5B–C), suggesting that the T6SS may stimulate IL-6 and IL-1β production independently of TNFα induction. Not surprisingly, IL-17, known to be induced by IL-1β, was also more up-regulated in RB50 stimulated macrophages compared to macrophages stimulated by the *clpV* mutant (Figure 5D). Stimulation of IL-6, IL-1β, and IL-17, independent of ClpV-mediated TNFα production, suggests that the T6SS may play a role in recruiting immune cells to the site of infection. We also investigated production of the anti-inflammatory cytokine, IL-10, and its antagonist Th1 cytokine, IFN-γ. We observed that wild type bacteria induced more IL-10 production than the *clpV* mutant and very low levels of IFN-γ, suggesting that a mutant lacking *clpV* may affect cell recruitment by altering IL-10 production (Figure 5E–F). Together, this data suggests that the T6SS may affect cytokine production that regulates inflammation initiation and cell recruitment, and may thereby affect downstream adaptive immune response pathways.

### T6SS is required for persistence in the murine respiratory tract

**Figure 6:**
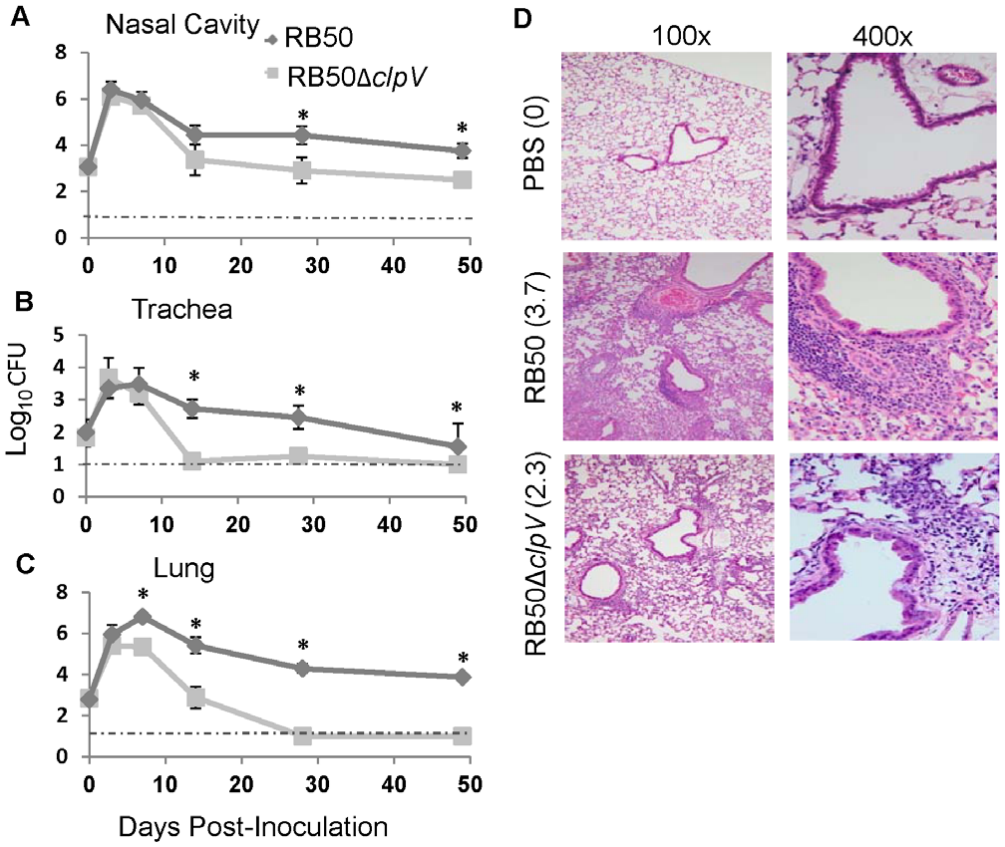
ClpV mediates pathology and persistence *in vivo*. A–C. Colonization of RB50 verses RB50Δ*clpV* in C57BL/6 mice at an inoculation dose of 5×10^5^ CFU in 50 µL in the nasal cavity (A), trachea (B), and lung (C). D. Representative H&E lung sections from C57BL/6 mice on day 3 post-inoculation and their average pathology scores.

To determine whether ClpV-dependent effects on cytokine production *in vitro* contribute to *in vivo* colonization and persistence of *Bordetella,* we used a well established murine model of infection [37], [63]. We inoculated C57BL/6 mice with 5×10^5^ CFU of *B. bronchiseptica* strain RB50 or RB50Δ*clpV*, and bacterial numbers were determined in the nasal cavity, trachea, and lungs at 0, 3, 7, 14, 28 and 49 days post-inoculation (Figures 6A–C). RB50Δ*clpV* colonized the respiratory tract similarly to RB50 for the first three days post-inoculation. However, by day 7 RB50Δ*clpV* numbers in the lungs were one tenth that of RB50 (Figure 6C). Compared to wild type, fewer RB50Δ*clpV* bacteria were reported in the trachea by day 14, and by day 28 numbers of the mutant were lower in the nasal cavity (Figures 6A and 6B). The bacterial load of both mutant and wild type declined over time in the nasal cavity, trachea, and lungs; however, RB50Δ*clpV* was cleared from the trachea and lungs 28 days post-inoculation, while significant numbers of RB50 could still be detected in the lungs 49 days post-inoculation. Wild-type *B. bronchiseptica* is known to persist indefinitely (>150 days) in the nasal cavities of laboratory mice at levels greater than 10^3^ CFU [64]. While RB50Δ*clpV* was still present in the nasal cavity after 49 days, it was reduced to approximately 10^2^ CFU (Figure 6A). Together, these data suggest that the T6SS contributes to the ability of *B. bronchiseptica* to persist in the murine respiratory tract.

### T6SS mediates increased pathology and cell recruitment *in vivo*

When mice were dissected three days following infection with RB50, their lungs were visibly inflamed and erythematous. In contrast, the lungs of mice infected with RB50Δ*clpV* appeared healthy (data not shown). We hypothesized that although bacterial loads recovered from each group were comparable at this time point, T6SS activity was causing enhanced leukocyte recruitment into the lungs and increased tissue damage and host cell necrosis. Histological analysis of lungs stained with H&E revealed significantly attenuated inflammatory pathology in mice infected with RB50Δ*clpV* (2.3 score) relative to those infected with RB50 (3.7 score) (Figure 6D). In the lungs of RB50 infected mice, we observed a robust accumulation of polymorphonuclear cells (PMNs) cuffing the perivascular spaces, infiltrating into the connective tissue underlying the respiratory epithelium of the bronchioles, and collecting within alveolar spaces. In comparison, lungs from mice infected with RB50Δ*clpV* had visibly reduced cellular infiltration into perivascular spaces and very little infiltrate in the alveolar spaces. Despite significantly higher pathology scores, a similar amount of necrotic cell death was observed in both the RB50 and RB50Δ*clpV* infected lungs (Figure 6D). The LD50 of wild type RB50 in C57BL/6 mice is approximately 10^6.3^ CFU, and fatality from this dose occurs within three days of inoculation [35]. C57BL/6 mice inoculated with up to 10^8.1^ CFU of RB50Δ*clpV* survived for at least 90 days, indicating the virulence of *B. bronchiseptica* requires this T6SS gene (data not shown). These data suggest that the T6SS contributes to *B. bronchiseptica-*mediated pathology and decreases the mean lethal dose of *B. bronchiseptica* strain RB50.

### T6SS modulates a Th1 immune response

**Figure 7:**
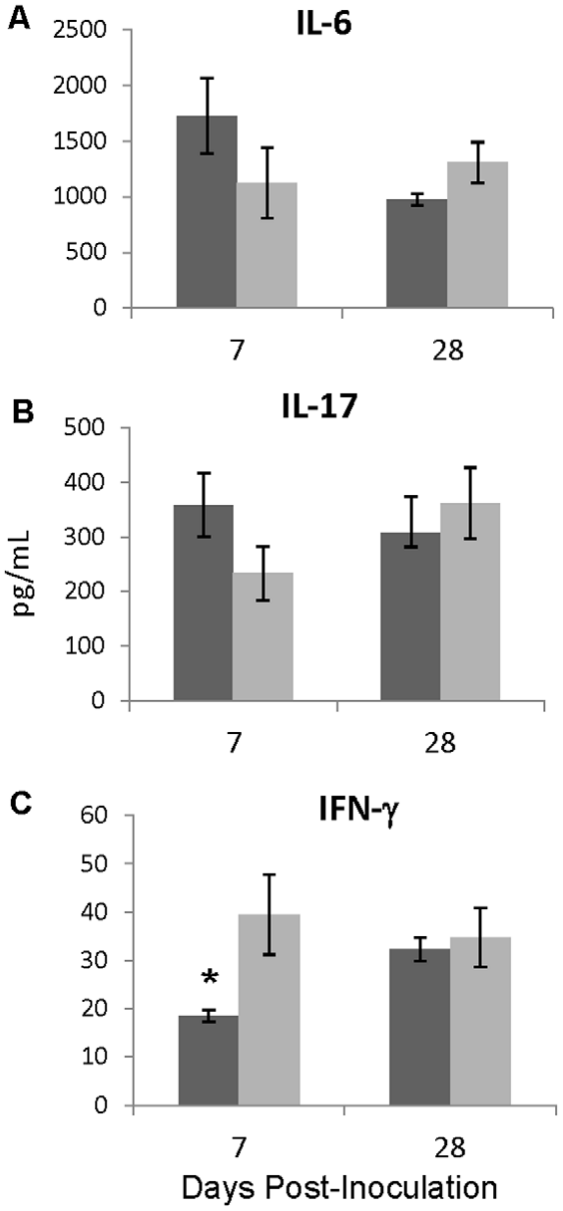
*In vivo* IFN-γ production is ClpV dependent. A–C. Cytokines recovered from lungs of mice infected with RB50 (dark bars) or RB50Δ*clpV* (light bars) for IL-6 (A), IL-17 (B), and IFN-γ (C) on days 7 and 28 post-inoculation. * denotes p value<0.05.

We observed decreased cytokine production *in vitro* and decreased cell recruitment *in vivo*. Therefore, we measured cytokines at the infection site to determine whether a functional T6SS alters cytokine production in ways that might cause skewing of the T helper cytokine response profile. We directly assayed lung homogenates from day 7 and 28 post-inoculation for the presence of cytokines. On day 7 post-inoculation, we found that mice infected with RB50Δ*clpV* had lower levels of cytokines associated with Th17 responses, IL-6 and IL-17, and significantly higher levels of the Th1 cytokine, IFN-γ, when compared to RB50 infected mice (Figure 7A–C). However, these differences were not observed in lung homogenates from day 28 post-inoculation, and no differences were observed between RB50 or RB50Δ*clpV* infection in TNFα, IL-1β, and IL-10 at either time point (data not shown). Th1 responses have been shown to be critical for immune mediated clearance of *B. bronchiseptica*, while Th17 cells have been shown to contribute to the clearance of closely related pathogen, *B. pertussis* [65]. These findings suggest that the T6SS may delay immune mediated clearance by shifting the immune reaction to a Th17 response and preventing the development of critical Th1 responses.

## Discussion

This work represents the first investigation of T6SS function in the genus *Bordetella* and describes a robust natural host infection model in which the subtleties of complex immune interactions with the host can be dissected. As with multiple other pathogens, we find that mutation of *clpV* affects macrophages cytotoxicity *in vitro* and that ClpV-dependent interactions with macrophages result in proteomic changes consistent with apoptotic responses. Additionally, we observed that ClpV contributes to IL-1β, IL-6, IL-10, and IL-17 production in murine macrophages *in vitro*, and using natural host infection, confirmed that changes in IL-17 and IL-6 production *in vivo* are ClpV-dependent. Enhanced immunopathology and respiratory tract persistence were also found to be ClpV-mediated, potentially by affecting the development of an effective Th1 immune response essential for the clearance of this pathogen. Our results suggest that *clpV*, a gene linked to T6SS function, is a novel virulence factor that significantly contributes to the pathology and persistence of the respiratory pathogen *B. bronchiseptica*.

Numerous Gram-negative pathogens, such as *Vibrio choleraee* [6], *Aeromonas hydrophila* [7], *Legionella pneumophila* [8], *Salmonella typhimurium*, and *Yersinia pseudotuberculosis* [66] possess a T6SS that contributes to virulence during *in vitro* infections. This study shows that the *B. bronchiseptica* T6SS gene *clpV* is required for macrophage cytotoxicity and identifies proteomic changes associated with T6SS-mediated cellular apoptosis, including proteins associated with intracellular bacterial survival (pyruvate kinase isozymes M1/M2) [67], intracellular signaling (voltage dependent anion selection channel) [68], structural mimicry (guanine nucleotide binding protein subunit beta 2) [69], and apoptosis (voltage dependent anion selection channel, transcription factor E2F7 and isocitrate dehydrogenase NADP fragment) [70]–[72]. Although the E2F family broadly contributes to eukaryotic cell cycle regulation, disregulation of E2F7 and E2F8 can lead to apoptosis in an E2F1-dependent manner [70]. Additionally, the *B. bronchiseptica* T6SS could be affecting host cell by targeting GTPases, as recently observed in *Burkholderia cenocepacia* [73]. It is currently unclear if the eukaryotic proteins identified here are directly controlled by bacterial factors or whether they are transcribed from downstream effects. The only bacterial protein identified in macrophages following RB50 infection was a NADH-quinone oxidoreductase (NQO) subunit C. In other Gram-negative pathogens, a six subunit complex including an NQO is used to transport sodium, catalyzing electron transfer from NADH to quinone [74]–[76]. Pathogens, such as *Helicobacter pylori,* have been shown to require similar reductases to withstand oxidative stress while inside phagocytic cells [77]. Alternatively, there is speculation that deregulation of NADH may be involved in eukaryotic programmed cell death, suggesting a pathogenic mechanism for this protein [78], [79]. Further research examining these discrete protein interactions will better elucidate the specific T6SS pathogenesis mechanisms.

It is likely that interactions between the *B. bronchiseptica* T6SS and macrophages mediate critical activities very early in the course of infection. Within the first three days of RB50 infection, although the colonization burden of mutant and wild type are equal, there is apparent T6SS-dependent immune-pathology and cell recruitment. Both *in vitro* and *in vivo*, ClpV contributed to IL-1β, IL-6, and IL-17 production, potentially explaining the heightened cellular recruitment to the lungs. Thus, increased lung leukocytes recruitment in mice infected with RB50 might be predicted to lead to more rapid clearance of RB50; however, reduced numbers of RB50Δ*clpV* were recovered from lungs as early as 7 days post-inoculation. This increased immunopathology correlates with lower *in vivo* production of T-helper 1 (Th1) cytokines, such as IFN-γ. Antibody production and Th1 responses have been shown to be essential for *B. bronchiseptica* clearance *in vivo*, and it has been hypothesized that *B. bronchiseptica* evolved to stimulate IL-10 production to evade clearance [80], [81]. RB50Δ*clpV* stimulates a robust Th1 response, which likely contributes to its increased clearance from the lower respiratory tract. The exact mechanisms behind *B. bronchiseptica-*induced pathology remain unclear, although this could be attributed to a T6SS mediated cytotoxicity toward macrophages and a subsequent inflammatory response. Although not fully understood, heightened immune-pathology and increased bacteria numbers may enable bacteria to cause disease symptoms, such as coughing that may enhance transmission. Alternatively, localized pathology may facilitate initial colonization by inducing inflammation that disrupts mucociliary clearance mechanisms or resident host microflora.

*B. bronchiseptica* has previously been observed to persist indefinitely in the nasal cavity of experimental mice [35], [63], [82]; however the *clpV* mutant persists at much lower levels. It is unclear whether the T6SS enables long-term persistence at higher numbers in the nasal cavity by modulating key early immune interactions that subvert productive adaptive immune responses or whether T6SS mediates ongoing resistance to opsonophagocytic clearance. Recent work showed that *P. aeuroginosa* toxin, Tse2, part of a toxin-immunity system secreted through the T6SS, mediates killing of other prokaryotic organisms, but not eukaryotic organisms [58]. Since then, bacteriocidal activity has also been observed in *Vibrio* and *Serratia* species [52], [83]. Although we have not been able to identify any obvious Tse2 homologs in the *B. bronchiseptica* genome to date, the T6SS may mediate protection or confer an advantage over host nasal microflora, preventing its displacement by competitor species. Interestingly, the Mekalanos laboratory recently determined that *clpV* is required *V. cholerae* virulence against amoebae but is not as important for T6SS-dependent bacterial killing, suggesting that ClpV may have multiple functions that contribute to persistence in the host [52], [53], [88].

Of the three classic *Bordetella* strains that have been sequenced, *B. bronchiseptica* strain RB50, *B. parapertussis* strain 12822 and *B. pertussis* strain Tahoma I, RB50 is the only strain whose T6SS is predicted to be functional. Previous work has shown that RB50 is also the only one of these strains known to be cytotoxic to macrophages; *B. pertussis* and *B. parapertussis* have been shown to be non-cytotoxic for up to six hours *in vitro* [21]. Strikingly, RB50Δ*clpV* cytotoxicity is similar to that of *B. pertussis* and *B. parapertussis*. Although *Bordetella* cytotoxicity has been attributed to ACT and the T3SS, the loss of T6SS function may explain why *B. pertussis* and *B. parapertussis* strains do not kill macrophages even though they express ACT and, in some cases, have a functional T3SS [24], [84], [85]. Surprisingly, RB50Δ*clpV*, which would be expected to retain T3SS-mediated cytotoxicity, killed less than 10% of macrophages even after six hours at an MOI of 1, suggesting that the T3SS and T6SS may have cooperative or synergistic effects. In *B. bronchiseptica*, there is significant overlap in the phenotypes of T3SS and T6SS mutants; both mutants display overall decreased pathology, shortened duration of colonization, and attenuated virulence *in vivo* [32], [34], [86], [87]. However, only the T6SS appears to be required for IL-6 production and nasal cavity persistence. Interestingly, the *B. bronchiseptica* T3SS is known to be BvgAS regulated, while microarray data from T. Nicholson *et al*. indicate that the T6SS is not regulated by this master regulatory system [40], suggesting that these secretion systems may be expressed and/or required under different circumstances. Notably, the T3SS and T6SS have been shown to be expressed at alternate times during *Pseudomonas* and *Salmonella* infection [10], [16], [60]. Further work is necessary to understand the interactions between these secretion systems and how they independently and cooperatively affect the infection process.

## Supporting Information

Table S1 **Primer sets for the deletion and confirmation of** ***clpV*** **removal from the** ***B. bronchiseptica*** **strain RB50 genome.**
(TIFF)

Table S2 **Quantitative real time primers for detecting** ***hcp*** **,** ***clpV*** **,** ***vgrG*** **, and** ***icmF*** **in** ***B. bronchiseptica*** **strain RB50.**
(TIFF)

Figure S1 **2D gel electrophoresis was completed on macrophages inoculated with RB50 (A) or RB50Δ** ***clpV*** **(B).** The black box indicates the portion of the gel that is enlarged in Figure 4.
(TIFF)

Figure S2 **A Gaussian version of the two 2D gel images, one containing supernatant from RB50 infected macrophages and the other from RB50Δ** ***clpV*** **infected macrophages, was created.** Red circles indicate proteins that were present only in the RB50 infected macrophages, but were absent in the RB50Δ*clpV* infected macrophages. Proteins chosen for identification are labeled and indicated by an arrow in the enlarged portion of the gel.
(TIFF)
