# Supplementary figures and images for: A Type VI Secretion System Encoding Locus Is Required for Bordetella bronchiseptica Immunomodulation and Persistence In Vivo
Source: PLoS One. 2012 Oct 12;7(10):e45892. doi: 10.1371/journal.pone.0045892 (PMC3470547; doi:10.1371/journal.pone.0045892)

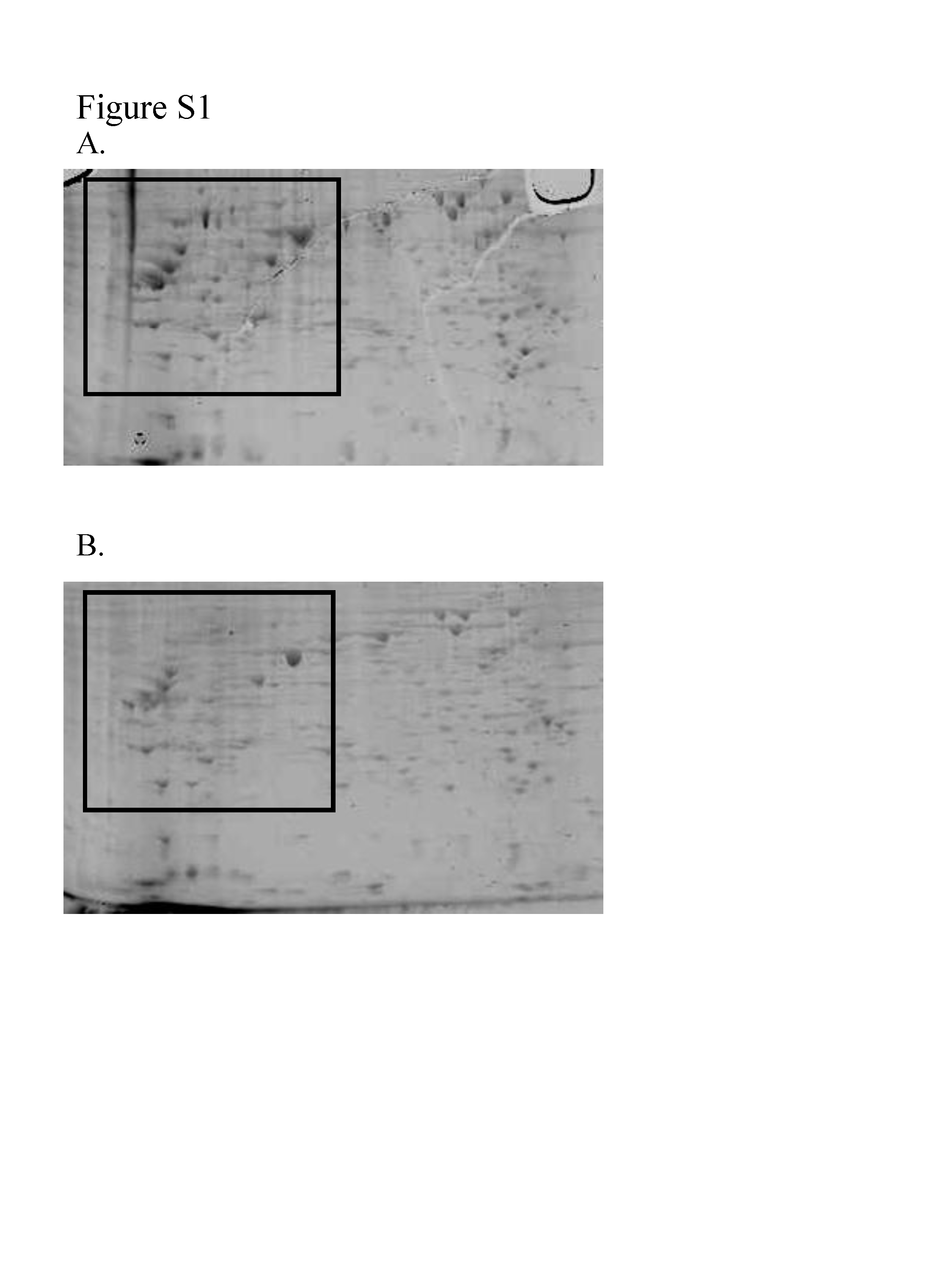

Supplement: Figure S1 — 2D gel electrophoresis was completed on macrophages inoculated with RB50 (A) or RB50Δ clpV (B). The black box indicates the portion of the gel that is enlarged in Figure 4. (TIFF) [file pone.0045892.s003.tif]

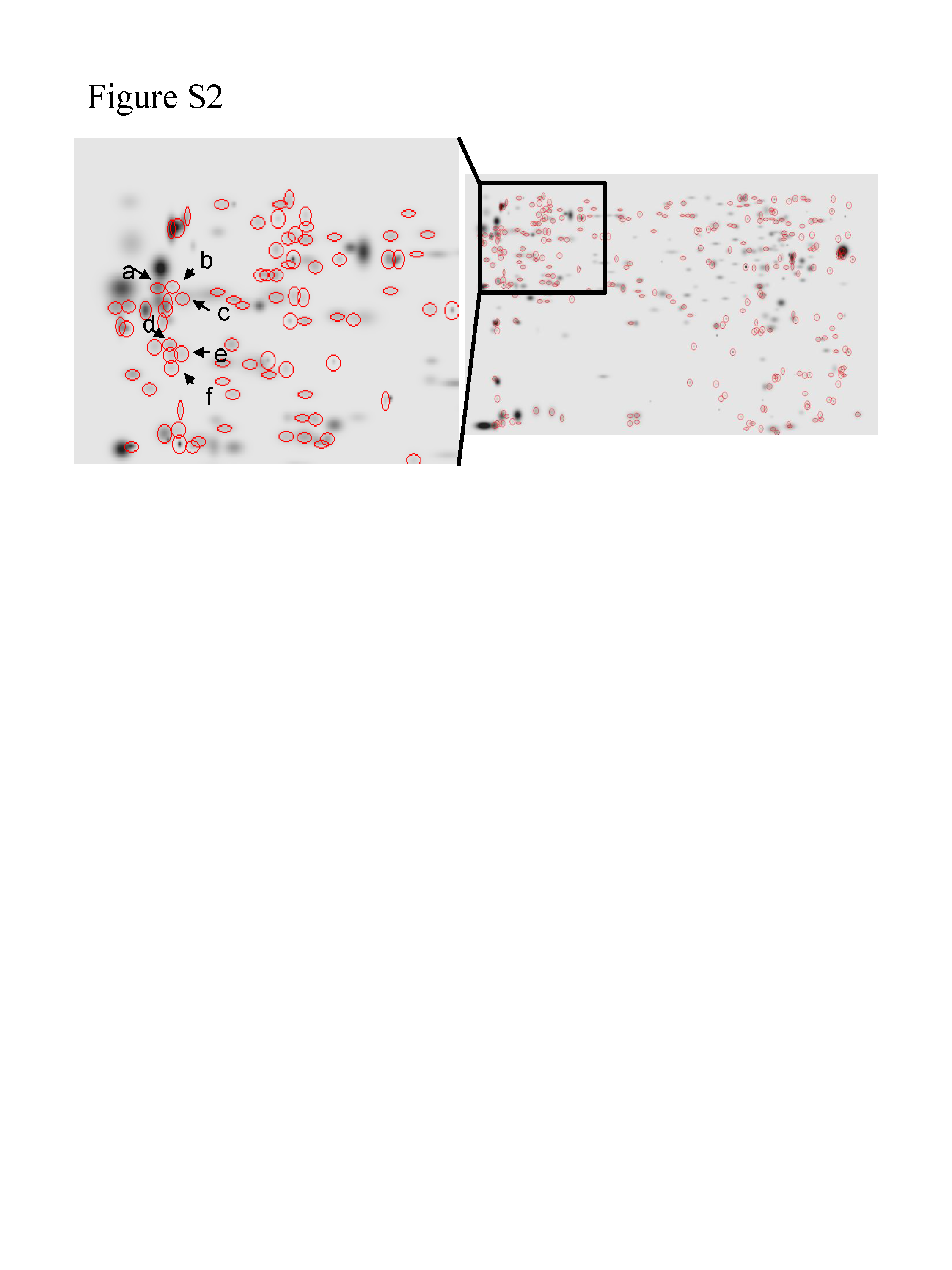

Supplement: Figure S2 — A Gaussian version of the two 2D gel images, one containing supernatant from RB50 infected macrophages and the other from RB50Δ clpV infected macrophages, was created. Red circles indicate proteins that were present only in the RB50 infected macrophages, but were absent in the RB50ΔclpV infected macrophages. Proteins chosen for identification are labeled and indicated by an arrow in the enlarged portion of the gel. (TIFF) [file pone.0045892.s004.tif]
